# Distinct signatures of lung cancer types: aberrant mucin O-glycosylation and compromised immune response

**DOI:** 10.1186/s12885-019-5965-x

**Published:** 2019-08-20

**Authors:** Marta Lucchetta, Isabelle da Piedade, Mohamed Mounir, Marina Vabistsevits, Thilde Terkelsen, Elena Papaleo

**Affiliations:** 1 Computational Biology Laboratory, Danish Cancer Society Research Center, Strandboulevarden 49, 2100 Copenhagen, Denmark; 2 Novo Nordisk Foundation Center for Protein Research, Faculty of Health and Medical Sciences, University of Copenhagen, Copenhagen, Denmark

**Keywords:** Lung adenocarcinoma, Lung squamous cell carcinoma, Differential expression analysis, RNA-Seq, Co-expression, Soft clustering, Survival analysis, TCGA

## Abstract

**Background:**

Genomic initiatives such as The Cancer Genome Atlas (TCGA) contain data from -omics profiling of thousands of tumor samples, which may be used to decipher cancer signaling, and related alterations. Managing and analyzing data from large-scale projects, such as TCGA, is a demanding task. It is difficult to dissect the high complexity hidden in genomic data and to account for inter-tumor heterogeneity adequately.

**Methods:**

In this study, we used a robust statistical framework along with the integration of diverse bioinformatic tools to analyze next-generation sequencing data from more than 1000 patients from two different lung cancer subtypes, i.e., the lung adenocarcinoma (LUAD) and the squamous cell carcinoma (LUSC).

**Results:**

We used the gene expression data to identify co-expression modules and differentially expressed genes to discriminate between LUAD and LUSC. We identified a group of genes which could act as specific oncogenes or tumor suppressor genes in one of the two lung cancer types, along with two dual role genes. Our results have been validated against other transcriptomics data of lung cancer patients.

**Conclusions:**

Our integrative approach allowed us to identify two key features: a substantial up-regulation of genes involved in O-glycosylation of mucins in LUAD, and a compromised immune response in LUSC. The immune-profile associated with LUSC might be linked to the activation of three oncogenic pathways, which promote the evasion of the antitumor immune response. Collectively, our results provide new future directions for the design of target therapies in lung cancer.

**Electronic supplementary material:**

The online version of this article (10.1186/s12885-019-5965-x) contains supplementary material, which is available to authorized users.

## Background

Lung cancer is one of the most aggressive cancers, with a five-year overall survival of 10–15% [1]**.** Lung cancer can be classified into small cell lung cancer (SCLC) and non-SCLC (NSCLC), which account for 15 and 85% of all lung cancers, respectively. The main subtypes of NSCLC are divided into adenocarcinoma (LUAD) and squamous cell carcinoma (LUSC). Lung cancer is a highly heterogeneous cancer type with multiple histologic subtypes and molecular phenotypes [2, 3].

Since 2015, the classification of lung tumors has been defined by cytology and histology [1, 4]. Despite the staining strategy to separate lung tumors into different classes, cases that are ambiguous at the immunohistochemical level are often reported and difficult to resolve. A proper differentiation between LUAD and LUSC determines eligibility for certain types of therapeutic strategies [5]. For example, some drugs are contraindicated for one of the two lung cancer types, such as Bevacizumab (Avastin) in LUSC [6]. It thus becomes crucial to discriminate among the two lung cancer types in a precise way.

Microarray technologies have been used to identify differentially expressed genes in lung cancer samples helping to pinpoint critical markers [7–10]. For example, Naval et al. identified a prognostic gene-expression signature of 11 genes, which was subsequently validated in several independent NSCLC gene expression datasets [9]. This pioneering study established the prognostic impact of changes in gene-expression for NSCLC patients. However, the markers identified in the study do not differentiate between LUAD and LUSC.

Gene or microRNA markers may be used, in principle, to distinguish between these two types of cancer [11–14]. In this context, single markers are unlikely to be sufficiently robust to discriminate between cancer subtypes due to the intrinsic heterogeneity of tumors. New methods have been developed for robust analyses of co-expression signatures in gene expression data [15–17]. Co-expressed modules are groups of genes that not only show high correlations of expression; they also encompass important information on the heterogeneity of phenotypes, cancer progression, or response to treatment [18–24]. We speculated that the integration of differential expression and co-expression analyses could be beneficial for the comparison of cancer (sub)types.

The Cancer Genome Atlas (TCGA) is a large genomic initiative in which more than 10 000 patients were profiled using six different platforms to identify cancer-related signatures [25–27]. TCGA provides a unique resource which can be re-analyzed for the discovery of cancer-related alterations or new biomarkers specific to certain cancer (sub)types. Among the next-generation sequencing (NGS) platforms available, RNA-Seq is a reliable approach for quantification of changes at the transcriptional level [28]. Lung cancer datasets for LUAD [29] and LUSC [30] are available in TCGA and account for more than 1000 samples overall. In parallel, the Recount2 initiative [31], which integrates GTEX (Genotype-Tissue Expression Project) [32] and TCGA, has recently allowed for an increase of healthy tissue samples for the comparison with tumor samples. Thus, the LUAD and LUSC TCGA datasets offer a suitable starting point for the identification of gene expression signatures that could discriminate between the two lung cancer types in terms of classification, diagnosis, and prognosis, along with to shed light on the underlying molecular mechanisms. These two TCGA datasets have been used either to identify general cancer signatures [10, 33–39] or to pinpoint signatures specific to only one of the lung cancer types [40–42].

Cline and colleagues [39] recently showed that there is a subset of 19 samples in the TCGA LUSC cohort that feature a LUAD-like gene expression profile. They labeled these samples ‘discordant LUSC’. Discordant LUSC samples are borderline for subtype classification, and the similarity with LUAD is also modest. These findings were supported by the analyses on an alternative pre-processing of the TCGA datasets [38]. As such, it is essential to account for this information in the re-analysis of the TCGA lung cancer data to avoid misleading conclusions.

**Fig. 1:**
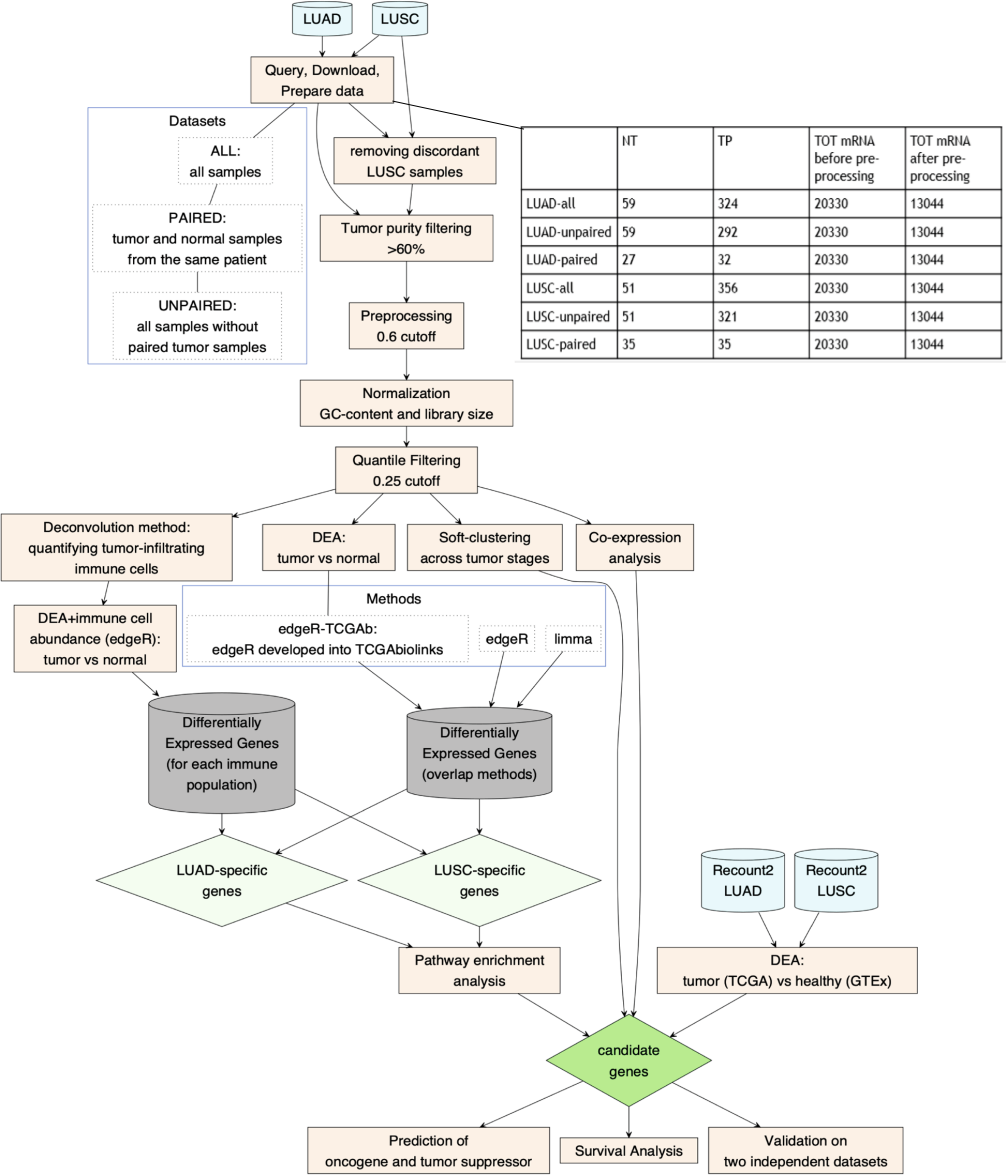
The workflow illustrates the steps used in our study. We used the R/CRAN package *DiagrammeR* v.1.0.1 to illustrate the workflow

We aimed to closely compare LUAD and LUSC TCGA datasets using a robust statistical and bioinformatic framework (Fig. 1). In particular, we: i) identified a group of genes that are differentially expressed between LUAD and LUSC when compared to the normal samples; ii) assessed changes in the gene expression signature over cancer stages; iii) identified modules of differently co-expressed genes in the two lung cancer types and alterations in their transcriptional regulation; iv) predicted potential oncogenes, tumor suppressors or dual role genes for each type and, iv) evaluated if any of the LUAD- or LUSC-specific candidate genes had a prognostic potential. Overall, our study resulted in a subset of genes and pathways that could be used to discriminate among the two cancer types. Moreover, we identified candidate genes which are suitable for further functional/structural studies since they are poorly understood and potentially important as lung cancer markers or targets. Our data also provide a useful guide for future cellular studies using cancer cell lines, which reflects the LUAD or LUSC types.

## Methods

### Pre-processing of RNA-Seq data from the Cancer genome Atlas (TCGA)

We downloaded and pre-processed level 3 legacy RNA-Seq data (RSEM count) for LUAD and LUSC with the *GDCquery* of the *TCGAbiolinks* Bioconductor/R package [43, 44]. The RNA-Seq data have been produced using the Illumina HiSeq 2000 mRNA sequencing platform.

We downloaded the data in October 2016 from the Genomic Data Common (GDC) Portal (https://portal.gdc.cancer.gov). An overview of the analyzed samples is reported in Fig. 1. We removed the 19 ‘discordant LUSC’ samples before analysis [38], along with samples with low tumor purity (< 60%) using a consensus measurement of tumor purity [45].

We then employed the *TCGAbiolinks* [43] function *GDCprepare* to obtain a *Summarized Experiment* object [46]. We removed outlier samples with the *TCGAanalyze_Preprocessing* function of *TCGAbiolinks* using a Spearman correlation cutoff of 0.6. We normalized the datasets for GC-content [47] and library size using the *TCGAanalyze_Normalization*. Lastly, we filtered the normalized RNA-Seq data for low counts across samples using the function *TCGAanalyze_Filtering*. This step removed all transcripts with mean across all the samples less than 0.25 quantile of the mean. The pre-processed and processed datasets are available through our *Github* repository, along with the script to generate them (https://github.com/ELELAB/LUAD_LUSC_TCGA_comparison).

### Differential expression analyses of TCGA datasets

Differential expression analyses have been carried out using *edgeR* [48] and *limma* [49]. The analyses were performed using three different pipelines. One pipeline was based on *limma*-*voom* and the other two were *edgeR*-based. One of the two *edgeR*-based pipelines was implemented in the *TCGAanalyze_DEA* function which was incorporated into the first release of the *TCGAbiolinks* package (called *edgeR-TCGAb* in this study for sake of clarity).

We applied the *voom* transformation to RNA-Seq data analysis with *limma* [50] to estimate precision weights for each count, before performing the differential expression analysis. In *edgeR* pipelines, we used the *GLM* (Generalized Linear Models) approach. Both the *limma* and *edgeR* pipelines allowed to include an experimental design with multiple factors.

In the *limma* and *edgeR* pipelines, the design matrix includes: conditions (tumor vs normal), the patient information when a paired dataset was used, and batches for the other datasets (*unpaired* and *all*). We corrected for the TSS (Tissue Source Site; the center where the samples are collected) as source of batch effect in the *edgeR* and *limma-voom* DEA pipelines. In contrast, *edgeR-TCGAb* implemented a simple function for DEA which did not account either for batch corrections or patient information. In all the DEA analyses, we defined as a cutoff a log fold change (logFC) > = 1 or < = − 1 to retain significant DE genes, along with a False Discovery Rate (FDR) cutoff of 0.01.

During the analyses, we tested two variations of the *limma-voom DEA* pipeline: I) using the same design matrix for *voom* and *lmFit* functions and ii) using the entire *voom* object in the steps following the *voom* transformation and not only the log2-transformed data. These adjustments provide a more correct approach to DEA but did not make any difference on our final conclusions. The corresponding scripts are also reported in our *Github* repository.

The overlap between the DE genes identified by each pipeline and for each different curation of the dataset was evaluated with the *UpSetR* package [50].

### Curation and differential expression analyses of unified GTEx and TCGA LUAD and LUSC datasets

We used the unified dataset integrating the GTEx [32] cohort of healthy samples and TCGA data as provided by the *Recount2* protocol [31]. We employed the *TCGAquery_Recount2* function of *TCGAbiolinks v2.8* to query the GTEx and TCGA unified dataset for lung cancer [51]. We filtered the data for tumor purity with a threshold of 60% as we did for the TCGA dataset and removed the LUSC discordant samples.

Since *recount2* barcodes were updated to the Universally Unique Identifier (UUID), we convert them to filtered TCGA barcodes with the *TCGAAutils* package, so that we could apply the pre-processing steps with *TCGAbiolinks*. The mapping between the TCGA barcodes and the new UUIDs was obtained by extracting the GDC case identifiers. We analyzed 374, 355 and, 393 samples for GTEx, LUAD and, LUSC, respectively in the unified datasets. After the filtering steps and preparation of the unified datasets for LUAD and LUSC, only protein-coding genes were retained using the *biomaRt* Bioconductor/R package [52–54]. We carried out GC-content normalization and quantile filtering as described above. We converted the ENSEMBL identifiers into gene names through the information in the *SummarizedExperiment* object. The DEA was carried out with the *limma-voom* method according to the pipeline described in the previous section.

### Analysis of the tumor microenvironment

To appreciate the differences between LUAD and LUSC immune landscapes, we estimated the abundance of populations of tissue-infiltrating immune cells using the R package *MCP-counter* [67]. We used the *MCPcounter.estimate* function to estimate the abundance of immune cell populations (T cells, cytotoxic lymphocytes, B cell and, monocytic lineage, myeloid dendritic cells, neutrophils) and non-immune stromal populations (endothelial cells and fibroblasts) for each sample. In particular, the gene matrix obtained after the pre-processing steps was used as input for the analysis.

For each cell population, we divided the samples into four groups (see Github repository): i) very-low (population from 0 to the minimum value of the distribution); ii) low (from the minimum to the first quartile); iii) medium (from the first to the third quartile); and iv) high (from the third quartile to the maximum value) abundance. We incorporated this information into the design matrix for DEA with *edgeR* to identify the DE genes for each cell population and each lung cancer type in comparison with the normal samples. These different sets of DEAs were compared to the consensus DEA for the selection of the candidate genes and to assess the robustness of the pathway-enrichment and GO-enrichment analyses (see Pathway enrichment analyses Section).

### Soft-clustering analysis

We performed gene clustering for the LUAD and LUSC datasets *paired* and *all* according to the normal tumor (NT) and four clinical stages of cancer, i.e., stages I, II, III, and IV using the *Mfuzz* package version 2.36.0 [55]. *Mfuzz* uses a fuzzy c-means algorithm based on the iterative optimization of an objective function to minimize the variation of objects within the clusters. The fuzzy c-means algorithm is robust to noise and avoids a priori pre-filtering of genes [56]. We assessed the longitudinal evolution of the mean of expression in the LUAD and LUSC clusters in the normal samples and along the four stages of tumor progression.

The dataset with all the samples were used for the soft-clustering analyses and a consensus matrix containing all the gene expression for LUAD and LUSC was built. We collected all barcodes corresponding to NT samples using the *TCGAbiolinks* function *TCGAquery_SampleTypes* for each lung subtype to identify NT samples in the gene matrices. We mapped the tumor samples to their stages from the LUAD and LUSC clinical datasets using their barcodes. We filtered out the samples with “not reported” stage status. We computed the mean gene expression value per tumor stage and NT for LUAD and LUSC. We defined a maximum number of clusters equal to six for the fuzzy c-means clustering. The centroid clustering step results from a weighted sum of all cluster members and shows the overall gene expression pattern in each cluster. The membership values indicate how well a gene is represented by its cluster. Low values illustrate a poor representation of the gene by the cluster centroid. Large values point to a high correlation of the expression of the gene with the cluster centroid. In each cluster, genes are represented with color lines corresponding to their cluster membership m > 0.56. We selected this cutoff empirically to be stricter than the default value of 0.5. The membership values are color-encoded in the plots generated by *mfuzz.plot*.

### Pathway enrichment analyses

We used the *ReactomePA* version 1.18.1 R/Biconductor package to perform the Reactome-based Pathway Analysis [57]. We employed the *enrichPathway* function of *ReactomePA* to retrieve the enriched pathways in the DE gene set or in the list of genes from the soft clustering. We used the entire gene matrix after pre-processing as a background. An adjusted *p*-value cutoff of 0.05 was set, and the analysis was done by separating the up- and down-regulated genes for each dataset (*all* and *paired*) and lung cancer subtype. In addition, all the gene symbols were converted to their corresponding ENTREZ IDs provided by the *SummarizedExperiment* object (*GDCprepare* function output). We used *clusterProfile* [58] to illustrate the results of the pathway enrichment analyses.

### Gene ontology enrichment analysis

To identify biological functions in LUAD and LUSC DE gene sets, we carried out a Gene Ontology (GO) classification, which included the following categories: biological process, cellular component and molecular functions [59].

We performed GO functional enrichment analysis for the DE gene set using the *topGo* R/Bioconductor package. We provided both DE and background genes lists separating up- and down-regulated genes. We used the same background used in the Pathway enrichment analyses. The GO results for the biological processes were represented in circular plots with the *GOplot* R package [60].

### Co-expression network analyses

We used the LUAD and LUSC datasets upon filtering and after *voom* transformation (see Differential expression analyses of TCGA datasets section) to carry out modular co-expression analyses with the *CEMiTool* Bioconductor/R package [15] using the protocol suggested by the developers. We performed pathway enrichment analyses and protein-protein network analyses with the pre-built functions of *CEMiTools.* As a reference for protein-protein interactions, we used the *Interologous Interaction Database I2D* version 2.9. [61].

### Survival analysis

We performed a survival analysis using the *survival* R package version 2.41–3. We used Cox regression [62] to estimate differences in survival between patients with low and high expression levels of our candidate genes. For each cancer type, tumor samples were extracted and separated by gene expression levels according to lower and upper percentile (25th and 75th, respectively). If the gene expression level of a specific gene in a certain sample was lower than the 25th percentile, the corresponding sample was labeled as *low*. Samples with the gene expression level greater than the 75th percentile were labeled as *high*. In cases of tumor duplicates (i.e., tumor samples from the same patient), we used the mean for the analysis. The clinical data were downloaded using the *GDCquery_clinical* function of *TCGAbiolinks*. We used only barcodes for which information regarding the last follow-up or death time of the patient was available.

Cox regression analyses were performed using the *coxph* function. Cox regression allows to account for additional explanatory variables, such as age at diagnosis, gender, and tumor stage. Before performing Cox regression, we tested the proportional assumption using the *cox.zph* function, and we retained only the genes which satisfy this test (11 and 13 genes for LUAD and LUSC, respectively). The *p*-values of each variable were corrected using the Benjamini and Hochberg (BH) method [63].

### In silico validation of the candidate genes on independent cohorts

To validate our candidate genes, we selected two microarray studies that include both LUAD and LUSC samples. The first study contains 139 and 21 samples for LUAD and LUSC, respectively [64]. The second dataset (GEO accession number: GSE33532) is composed of ten and four samples for LUAD and LUSC, respectively [65]. At first, the probe sets were converted to gene names using the *gconvert* function of the *gProfileR* package version 0.6.6 [66] and the non-converted probes were removed. Since multiple probe sets can identify the same gene, we collapsed them to obtain unique matches with the *collapseRows* function implemented in the *WGCNA* package version 1.63 [16]. We performed hierarchical clustering with the complete method and euclidean distance and visualized the results as heatmap with the *heatmap.2* function in the *gplots* package version 3.0.1.

## Results

### Curation and description of the datasets used in the study

The datasets for our analyses were curated to remove the LUSC discordant samples, along with samples with a low tumor purity and outlier samples with correlation lower than 0.6. The number of samples and genes retained for the analyses are reported in Fig. 1.

We performed differential expression analyses (DEAs) to identify a subset of differentially expressed genes in the two TCGA lung cancer datasets LUAD and LUSC tumor primary (TP) with respect to the normal (NT) samples. A clear consensus on the best DEA approaches for RNA-Seq data does not exist yet. Different DEA methods could provide different results [49, 68–71]. We employed three pipelines for DEA of LUAD and LUSC and generated a consensus list of DE genes (see Method section).

In addition, we curated three different datasets (*paired*, *unpaired,* and *all*) for each cancer type and the results are discussed in the Additional file 1: Text S1. A comparison of the DE genes obtained from the analysis of each of the three datasets allowed us to evaluate the impact of the different curations in terms of sample size or sample pairs.

### The usage of a too simplistic design in the DEA protocol has marked effects on the DEA results

**Fig. 2:**
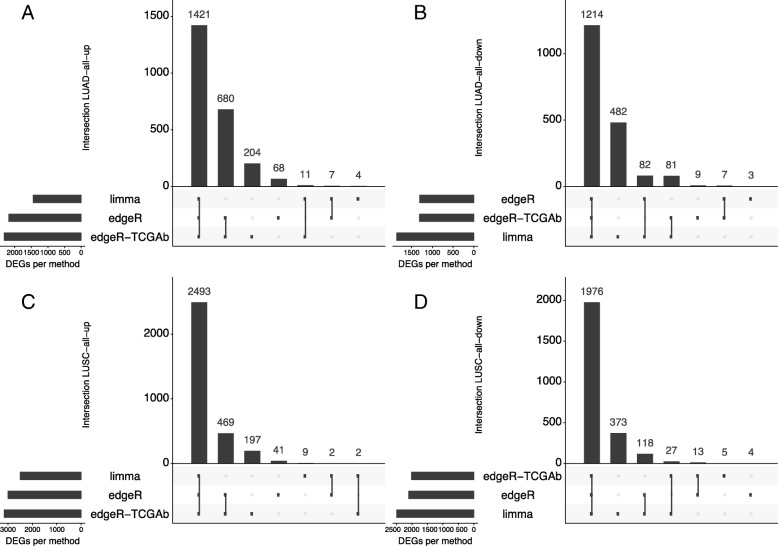
Comparison of DEA results with different DEA protocols. For sake of clarity, we reported the up-regulated genes (**a**,**c**) and down-regulated genes in (**b**,**d**) in LUAD (**a**,**b**) and LUSC (**c**,**d**), respectively (see https://github.com/ELELAB/LUAD_LUSC_TCGA_comparison for the whole set of results). The analyses have performed with the R/CRAN package *UpSetR* v.1.3.3

At first, we assessed the influence of using different definitions of the dataset for DEA analyses. We compared the results of DEA carried out with a certain method, i.e., *limma* or *edgeR* or *edgeR-TCGAb*, on the three different datasets (*paired*, *all*, and *unpaired*) for each of the two cancer types (LUAD and LUSC) (Figs. 1 and 2, and Additional file 1: Text S1). In our LUAD analyses, we used the *paired* dataset with 32 tumor and 27 normal samples, along with the *all* dataset with 324 tumor and 59 normal samples. For LUSC, the *paired* dataset contained 35 tumor and 35 normal samples, and the dataset *all* 356 tumor and 51 normal samples. In both cases, the *paired* datasets were small subsets of the corresponding full datasets. According to the comparison described in Additional file 1: Text S1, we focused on the dataset with all the normal and tumor samples for the following analyses.

*Limma* resulted in the most stringent approach for up-regulated genes (Additional file 5: Table S1, Fig. 2a-c). Inversely, *limma* provided a large number of down-regulated gene (more than 300), which were not identified by the *edgeR* pipelines (Fig. 2b-d).

*EdgeR-TCGAb* featured a subset of up-regulated genes which were not identified by the other two methods (Fig. 2a-c). In the case of paired samples, this behavior can be explained by the fact that the *edgeR-TCGAb* pipeline does not correct for patient-specific effects, which are likely more important when the normal and tumor samples are matched. Moreover, *edgeR-TCGAb* DEA pipeline does not include batch corrections, which we included within the design matrix in the other two DEA pipelines. We had a closer look at the 820 and 619 up-regulated genes identified only by the *edgeR-TCGAb* pipeline in LUAD and LUSC (Additional file 2: Figure S1). Most of the discordant genes have either logFCs close to or below 1 or FDR values close to 0.01. Moreover, *edgeR-TCGAb* tends to overestimate the logFC values. We also noticed that there was a small number of cases in which *edgeR-TCGAb* assigned an opposite directionality, i.e., the genes were down-regulated according to the other two methods. Specifically, this set of genes included: DUOXA2, IGFALS and, KLK14 in LUAD, as well as EDN3, GFI1B, MYH15, PEG3, and PENK in LUSC. We searched for each of the non-congruent genes in the *IGDB.NSCLC* database [72], which is a collection of genes that are altered in NSCLC. We did not consider hits for which the probe sets were reported with mapping problems or the fold change was lower than 2. We observed that only MYH15 was up-regulated in one of the LUSC studies at *IGDB.NSCLC*, while the other genes listed above were down-regulated, supporting the *edgeR* and *limma* results from our study.

The results of our analyses, thus, raised concerns about the accuracy of the original *TCGAbiolinks DEA* pipeline with *edgeR* (*edgeR-TCGAb*) especially when paired samples are analyzed, highlighting a need for a different DEA design within the R/Bioconductor package. This design should include functions for proper batch corrections and corrections for patient-specific effects, which we recently implemented in *TCGAbiolinks* [51]. Overall, 60–80% of the DE genes are in common among the three DEA methods, suggesting that their integration may allow for the removal of genes with borderline results to define a robust signature of LUAD- and LUSC-specific genes.

### Identification of LUAD- and LUSC-specific differentially expressed genes

We selected the datasets containing all the samples to maximize the sample size. As an additional control of our analyses, we carried out DEA on the LUAD and LUSC *unified* datasets from the recent *Recount2* initiative [31]. In the *Recount2* platform, TCGA data were integrated with the normal GTEX [32] samples. This integration increases the pool of available normal samples for the comparison for a total of 374 healthy samples. Moreover, *Recount2* provides a genuine source of healthy tissue samples to compare with lung tumors, e.g., not only the normal adjacent tissues that are available in the TCGA. The list of DE genes for LUAD and LUSC for the *unified* datasets are reported in our *GitHub* repository.

We employed a consensus approach, in which we defined as DE genes in LUAD and LUSC only those found by all the three DEA approaches (i.e., the intersects in each of the overlap diagrams similar to the ones reported in Fig. 2). We then compared the up- and down-regulated genes in LUAD with the ones of LUSC. To identify gene signatures that can differentiate between the two lung cancer types, it is not sufficient that the genes are differentially expressed with respect to the normal samples. One also needs to verify that they are not up (or down) regulated in both the cancer types.

We retained 337 and 1451 genes up-regulated, as well as 165 and 956 down-regulated genes in LUAD and LUSC, respectively (reported in our Github repository).

To verify that some of the differences observed for LUAD and LUSC DE genes did not come from differences in the composition of the tumor microenvironment between the two cancer types, we carried out additional 18 DEAs. In these DEA, we corrected for the populations of the different cellular infiltrations, which have been estimated using a deconvolution method (see Analysis of the tumor microenvironment section and Github repository for the lists of DE genes in different comparisons).

Interestingly, we identified a small subset of genes which were up-regulated in LUAD but down-regulated in LUSC (MUC5B, HABP2, MUC21, and KCNK5) or vice-versa (CSTA, P2RY1, and ANXA8) in all or the majority of the DEA comparisons.

We performed pathway enrichment analysis for the up- and down-regulated unique genes of LUAD and LUSC from the consensus DEA comparison, along with for each of the DEA analyses correcting for infiltration from other cellular populations. We only retained the genes and pathways common to the different DEA calculations. The analyses revealed that pathways related to O-linked glycosylation of mucins was enriched for the up-regulated genes of LUAD and down-regulated genes of LUSC, respectively (Table 1). This suggests that the proteins involved in this pathway could play an important role in discriminating between the two lung cancer types. Of particular interest is a group of mucins (MUC5B, MUC15, MUC16, and MUC21), along with enzymes involved in their modifications. Additionally, we noticed that genes involved in the complement system (C3, C5, CD55, and CFI) were down-regulated in LUSC. GO-enrichment analysis on LUAD DE genes also confirmed the importance of up-regulation of processes related to O-linked glycosylation, along with cellular adhesion (Additional file 3: Figure S2).

**Table 1:** Pathway enrichment analysis with *ReactomePA.* Only the results relevant to the comparison of O-glycosylation, immune response and complement pathways are reported. For the full list of results, one could refer to our Github repository for the project. We reported only the pathways and genes identified by the initial consensus DEA and the DE genes from the DEA comparisons accounting for the tumor  microenvironment. The range of FDR values identified for that pathway from the different enrichment analyses are reported as a reference

Pathway ID	Description	FDR	Gene IDs
913709	O-linked glycosylation of mucins	0.007–0.04	LUAD (up): B3GNT6/MUC16/MUC21/MUC5B
913709	O-linked glycosylation of mucins	0.01–0.07	LUSC (down): B3GNT7/B3GNT8/GALNT10/GALNT5/MUC1/MUC15/MUC21/MUC5B/ST6GALNAC4
977068	Termination of O-glycan biosynthesis	0.01–0.07	LUAD (up):MUC16/MUC21/MUC5B
5173105	O-linked glycosylation	0.01–0.05	LUAD (up): B3GNT6/ MUC16/MUC21/ MUC5B
5173105	O-linked glycosylation	0.01–0.03	LUSC (down): B3GNT7/B3GNT8/GALNT10/GALNT5/MUC1/MUC15/ MUC21/MUC5B/ST6GALNAC4/THBS1
977606	Regulation of Complement cascade	0.0002–0.06	LUSC (down):C3/C5/CD55/CFI
166658	Complement cascade	0.0008–0.06	LUSC (down): C3/C5/CD55/CFI

### Clustering of genes in LUAD and LUSC across tumor stages

We aimed to identify a subset of specific and interrelated genes which, as an ensemble, could be more effective than single markers in discriminating between LUAD and LUSC. For this purpose, DEA alone is not sufficient. We, therefore, analyzed the molecular signatures both using soft-clustering approaches over the stages of tumor progression and implementing weighted co-expression analyses.

**Fig. 3:**
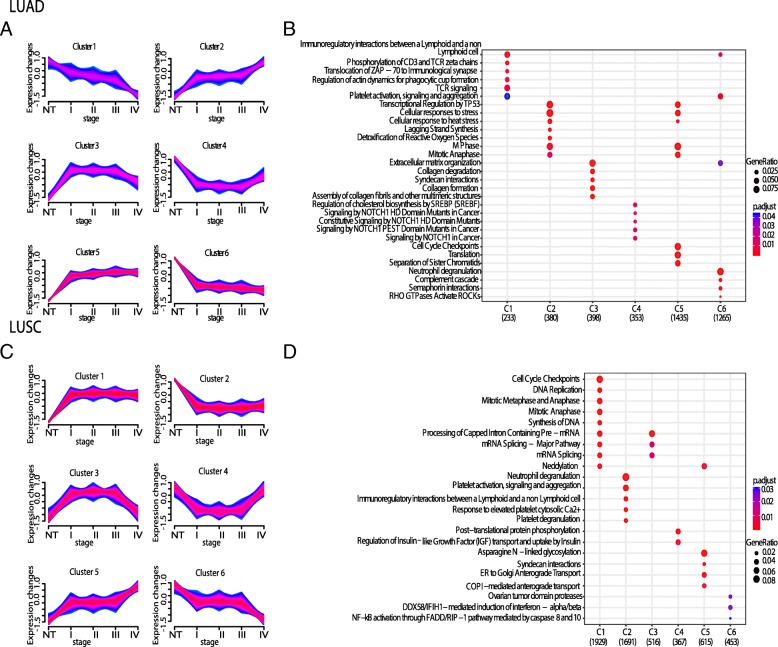
Soft-clustering across lung cancer stages of tumor progression. Each cluster describes an expression pattern in the dataset through the four stages of cancer i.e. stages I, II, III and IV. Blue and purple lines correspond to genes with high cluster membership value (i.e., m > 0.56). **a** Table with the genes belonging to each cluster and their m value is reported in our Github repository. The LUAD (**a**) and LUSC (**c**) clusters are showed along with the corresponding dotplots (**b** and **d**, respectively) for the top enriched pathways for each cluster. In the dotplots, the plots are colored according to the *p*-values from blue (high *p*-values and low enrichment) to red (low *p*-values and high enrichment). The cluster plots have been generated with the R/Bioconductor package *Mfuzz* version 2.36.0

We applied a soft-clustering approach [55, 56] to separate LUAD and LUSC genes into clusters based on their changes in gene expression in different tumor stages [73], allowing us to identify six clusters with different signatures (Fig. 3a-c).

Clusters 2 and 5 of LUAD (Fig. 3a), along with clusters 1 and 5 of LUSC (Fig. 3c) revealed a general up-regulation of genes along all stages. The two up-regulated clusters of genes in LUAD showed enrichment in transcriptional regulation of p53, cellular response to stress and, mitosis. In LUSC, mitosis was also up-regulated, together with other processes here among translation, cell cycle, ER, Golgi and COPI transports (Fig. 3b and d). In contrast, clusters 1 and 6 of LUAD (Fig. 3a), and clusters 2 and 6 of LUSC (Fig. 3c) featured a general down-regulation when the four tumor stages were compared to the normal samples, with no clear enrichment in biological pathways.

We extracted the genes that showed a trajectory of up-regulation across stages in one cancer type and down-regulation in the other, similarly to what we previously did for the DEA results. We identified a group of 46 genes which were up-regulated in LUAD but down-regulated in LUSC. 72 genes were down-regulated in LUAD and up-regulated in LUSC. The soft-clustering comparison provided an additional list of gene candidates of which MUC5B, CSTA, P2RY1 and, NTRK2 were shared between the soft-clustering and the DEA.

Clusters 3 of both LUAD and LUSC (Fig. 3a and c) featured a signature in which the genes were up-regulated at the early stages, but they decreased again at late stages (i.e. stage IV). Clusters 4 showed the opposite trend, i.e. a down-regulation at early stages but increase at late stages (Fig. 3a and c). These patterns may be indicative of dual-role genes [74]. The enriched processes were different for these genes in LUAD and LUSC. The dual-role of LUAD was associated with extracellular matrix organization, whereas in LUSC with mRNA splicing and mRNA processing (Fig. 3b and d). Expression of dual-role genes may, for example, be unwanted by cancer cells in early tumor stages, whereas the same genes become essential at later stages of tumorigenesis, providing the cancer cells with a functional advantage or resistance to chemotherapy [37].

### Prediction of oncogenes and tumor suppressor genes in LUAD and LUSC

Genes that are up- or down-regulated and are also known to be oncogenes or tumor suppressors, respectively, are of great interest in cancer.

We, therefore, carried out a prediction of potential tumor suppressor genes (TSGs) and oncogenes (OGs) using *Moonlight* [37], which employs gene expression signatures and biological pathways to identify potential TSGs and OGs. This analysis was used to integrate and expand the information available on TSGs and OGs through the curated data from *TSGene (TSGDB)* [75], *ONGene* [76], and *COSMIC* [77].

At first, we were interested in evaluating which of the up- and down-regulated genes that discriminate between LUAD and LUSC are known or predicted to be OGs (up-regulated genes) or TSGs (down-regulated genes).

We thus identified 24 potential TSGs and 146 OCGs for LUAD, while we obtained 22 TSGs and 456 OCGs for LUSC with *Moonlight*. The details and the full list of genes are reported in our *GitHub* repository. Only 31 predicted OCGs and no TSGs were common between LUAD and LUSC. Intriguingly, IL6 and KRT23 were predicted as OCGs in LUAD but TSGs in LUSC, highlighting that these genes could deserve attention in future studies. IL6 is of interest because of its role in the immune response and the complement system [78] and its down-regulation in LUSC. We recently identified IL6 as the only down-regulated cytokine in breast cancer samples using cytokine assays [79]. Future studies on naïve tumor samples or LUSC and LUAD cellular models, where the IL6 gene can be modulated by overexpression or silencing, could shed light on its role within the two lung cancer types [80].

### Co-expression signatures in LUAD and LUSC

As stated above, we sought gene expression signatures to discriminate between LUAD and LUSC types, or interesting targets for each cancer type. For this reason, we also carried out a gene co-expression analysis to identify different modular gene co-expression networks in LUAD and LUSC.

**Fig. 4:**
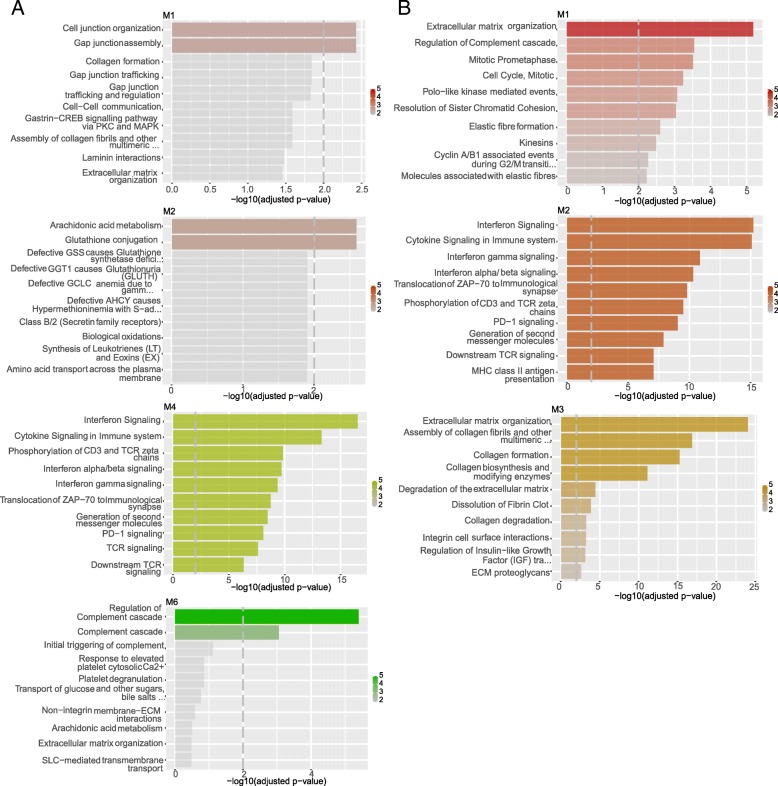
Co-expression modules and their network in LUSC (**a**) and LUAD (**b**). The modules which collect genes and pathways that differentiate LUSC from LUAD are shown in the figure. The analyses have been carried out with the R/Bioconductor package *CEMITools* version 3.10

In LUSC, we identified six modules (Fig. 4a). M1 was enriched in proteins for organization and assembly of the cell and gap junctions, including gap junction proteins, like the up-regulated hub proteins GJB5, keratin type II proteins and protein channels activated by chloride. M2 was enriched in proteins for glutathione conjugation and response to redox stress, such as the up-regulated hub proteins sulfiredoxin-1 protein and the oxidative stress-induced growth inhibitor OSGIN1. M3 included extracellular matrix organization and collagen-related proteins. M4 was enriched in interferon signaling, cytokine signaling in immune response, with a down-regulation of HLA genes. M5 was no associated with any annotated cellular pathway, whereas M6 was enriched in proteins that regulate the complement cascade.

We identified four modules in LUAD (Fig. 4b): M1 which was enriched in extracellular matrix organization proteins and regulation of complement cascade; M2, which was enriched in interferon and cytokine signaling and, M3 which included collagen-related genes and proteins for extracellular matrix organization. Notably, M3 was the only module that included hubs which were conserved among LUAD and LUSC. M4 of LUAD had no significant associations with any known pathway.

We noticed that some of the modules of LUAD and LUSC were enriched for the same processes. Nevertheless, a pairwise comparison of each of them suggested that, in most of the cases, the number of overlapping genes in the LUAD and LUSC modules is only a minor fraction. This could suggest that the genes triggering the same pathways had different co-expression signatures in the two cancer types. Pathway-enrichment analyses on DE genes or on the soft-clustering genes also pointed to a down-regulation of proteins involved in the complement cascade (Table 1) and genes related to the immune response in the LUSC samples (Fig. 3d), enforcing the notion of a compromised immune response in LUSC.

Moreover, the M1 and M2 of LUSC were enriched in pathways that have not been found for the LUAD co-expression modules, i.e., pathways related to cellular junctions (M1) and glutathione (M2).

**Fig. 5:**
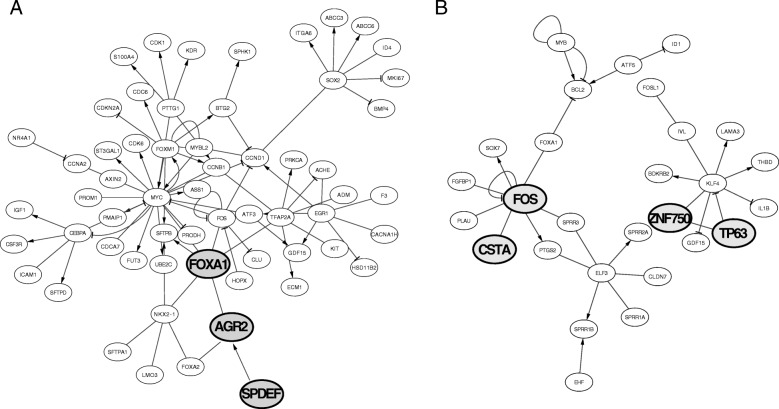
Network of transcription factors and their target genes in the co-expressed module 1 of LUAD (**a**) or LUSC (**b**). The data have been plotted using *Cytoscape* version 3.3.0

For further analyses, we retained only truly unique genes for LUAD or LUSC within each module. For each module, we extracted the known transcription factors and their targets using the *TRRUST* database as a source of information [81]. We identified a network of transcription factors and their targets for modules 1 and 2 of LUAD, as well as 1, 2,3, and 4 or LUSC (Fig. 5). Out of these, LEF1 was of interest since it can activate NRCAM. The two genes are not only co-expressed in the same LUSC module but also up-regulated in LUSC only. In module 1 of LUSC, we noticed the presence of the up-regulated CSTA, which is transcriptionally regulated by the FOS transcription factor, along with TP63 and its target gene ZNF750. In module 1 of LUAD, we identified an interesting network between the up-regulated gene AGR2 and its transcription factor FOXA1, along with the activator SPDEF.

### Selection of candidate genes and their pathways

We collectively considered the results of the analyses described above with the final goal of proposing a subset of LUAD and LUSC-specific genes for further studies. In particular, we decided to retain only the genes that satisfy the following criteria: i) genes that are up- or down-regulated in a specific cancer type and the opposite in the other cancer type according to the DEA analyses and/or soft-clustering analyses, and ii) genes that belong to the co-expression modules and are genuinely unique for LUAD or LUSC. For each of these genes, we also annotated information on their potential as oncogenes, tumor suppressors or dual role genes; known associations with cancer according to the repository of disease-gene associations from text mining of the literature *DISEASES* [82]. Specifically, we verified if they matched with known oncogenes or tumor suppressors through analyses of the *COSMIC* TGs and OCGs collection [77], *TSGDB* [75], *ONGene* [76] or prediction with the *MoonlightR* workflow [37]. For dual role genes, we integrated as a reference for our study the curation from *TSGDB* [75], *COSMIC* [77] and the recently predicted ‘double-agent’ genes, namely Proto-Oncogenes with Tumor-Suppressor Functions (*POTSF*) [74]. This integrative reference annotation for dual role genes is reported in Additional file 6: Table S2 and in the *Github* repository for a total of 152 genes, of which only 14 were all reported in all the three studies. The summary of each of the candidate genes and their annotations is reported in Table 2 and each candidate gene discussed in detail in the Discussion section.

**Table 2:** Candidate genes to discriminate between LUAD and LUSC in terms of gene expression levels, functions or prognosis

GENE	DEA	Mfuzz cluster	CEMiTool Module	DISEASES	OG	TSG
MUC5B	Up (LUAD) Down (LUSC)	Cl6(LUAD) Cl2(LUSC)	M1(LUAD)	None	None	None
HABP2	Up (LUAD) Down (LUSC)	Cl3(LUAD) Cl4(LUSC)	M1(LUAD)	3.8	None	None
MUC21	Up (LUAD) Down (LUSC)	Cl2(LUSC)	M1(LUAD)	4.0	None	None
KCNK5	Up (LUAD) Down (LUSC)	Cl3(LUAD) Cl2(LUSC)	M1(LUAD)	None	None	None
ICA1	Up (LUAD) Down (LUSC)^a^	Cl6(LUAD) Cl2(LUSC)	M1(LUSC)	1.9	None	None
CSTA	Down (LUAD) Up (LUSC)	Cl4(LUAD) Cl1(LUSC)	M1(LUSC)	3.5	None	TSGDB (CST5, CST6)
P2RY1	Down (LUAD) Up (LUSC)	Cl4(LUAD) Cl1(LUSC)	M1(LUSC)	2.2	Moonlight (LUSC, P2RY14) COSMIC(P2RY8)	None
ANXA8	Down (LUAD) Up (LUSC)	Cl5(LUSC)	M1(LUSC)	2.4	None	TSGDB (ANXA1, ANXA7)
FZD7	Up (LUSC)	Cl4(LUAD) Cl1(LUSC)	M2(LUSC)	3.7	ONGENE (FZD2) Moonlight (LUSC, FZD4)	None
ITGA6	Up (LUSC)^c^	Cl4(LUAD) Cl1(LUSC)	M1(LUAD)	4.2	ONGENE (ITGA3) Moonlight (LUSC, ITGA8)	TSGDB (ITGA5,ITGA7, ITGAV)
CHST7	Up (LUSC)^b^	Cl4(LUAD) Cl1(LUSC)	M2(LUSC)	1.6	None	TSGDB (CHST10)
ACOX2	Down (LUSC)	Cl2(LUSC)	M1(LUAD)	2.2	None	None
ALDOC	Up (LUSC)	Cl1(LUSC)	M1(LUAD)	3.9	None	None
AQP5	Down (LUSC)	Cl4(LUSC)	M1(LUAD)	None	None	None
ARSE	Up (LUAD)^a^ Down (LUSC)	Cl2(LUSC)	M1(LUAD)	None	None	None
FABP5	Down (LUAD) Up (LUSC)	Cl4(LUAD) Cl5(LUSC)	M1(LUSC)	None	MoonlightL (LUAD,FABP7)	TSGDB (FABP3)
SLC2A9	Down (LUAD)^a^	Cl4(DOWN) Cl5(LUSC)	M1(LUSC)	None	None	None
NRCAM	Down (LUAD)^a^ Up (LUSC)	Cl1(LUSC)	M2(LUSC)	2.6	Moonlight (LUSC, OCG)	TSGDB
AGR2	Up (LUAD) Down (LUSC)^b^	Cl3(LUAD)	M1(LUAD)	4.1	None	None
SPDEF	Up (LUAD) Down (LUSC)^b^	Cl1(LUAD) Cl2(LUSC)	M1(LUAD)	3.6	None	None

**‘**N.S.’ indicates not significant results. The *DISEASES* Z-score are provided in the table. ^a^, ^b^, and ^c^ indicate a significant DE when the unified recount, the TCGA pair dataset or both are used. We did not find any predicted dual-role genes for any of the candidate genes

### Association of the gene signatures with patient survival

Next, we aimed to evaluate if any of the candidate genes had a potential prognostic impact. We carried out survival analyses using a Cox proportional hazard regression with all the candidate genes. We accounted for different explanatory variables, including the clinical stages, age, and sex of the patients.

We assessed if a difference in the gene expression level (high or low) of the candidate genes could affect the survival rate of the patients. The unique genes with FDR values lower than 0.05 were ITGA6 and FABP5 in LUAD and ICA1 in LUSC (Additional file 7: Table S3). In details, these genes have an FDR associated with the ‘group_low’ less than 0.05. In particular, the hazard ratio (the exp. (coef)) is around 0.4 for the three genes. This means that a patient who has a high level of expression of one of the three genes is 0.4 times as likely to die at any time than a patient with a low level of expression of the same gene. Therefore, the risk associated with high gene expression of ITGA6, FABP5 (in LUAD) or ICA1 (in LUSC) is low, resulting in a better prognosis for these patients.

### Analysis of the candidate genes on independent datasets

To further strengthen our results, we validated the most interesting markers using two independent datasets, where LUAD and LUSC samples were profiled by transcriptomics techniques with the same experimental setup.

**Fig. 6:**
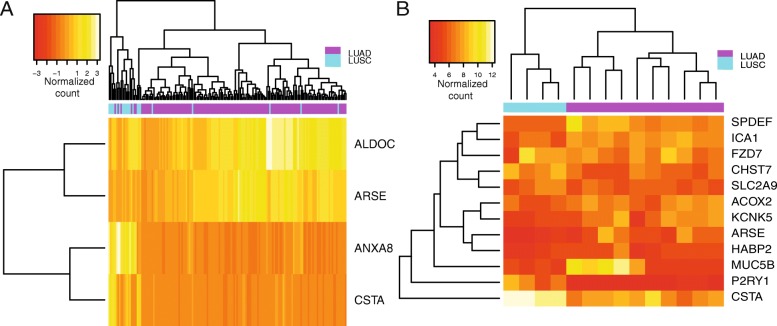
In silico validation of the candidate genes using other transcriptomics data for lung cancer. The figure shows the groups of candidate genes that were able to separate LUAD and LUSC samples selected by the two microarray studies used for validation. Panel **a** is related to the first study (see also Additional file 4: Figure S3), panel **b** to the second dataset (see In silico validation of the candidate genes on independent cohorts section for more details). The figure has been generated with R/CRAN package *gplots* version 3.0.1.1

For this analysis, we retained the candidate genes reported in Table 2 for which LUAD’s and LUSC’s upper and lower quartiles were sufficiently separated when compared for the same gene so that they may suggest a potential value as a marker for classification of the two lung cancer types. We then extracted the ones for which gene quantification was available in the validation datasets, and we used unsupervised clustering to verify if they could separate LUAD and LUSC. The results are reported in Fig. 6 and Additional file 4: Figure S3. We could not validate the clustering potential of mucins (except for MUC5B) since the probes for these genes were not available upon the probe set collapse step. MUC5B, HAPBP2, SPDEF, ICA1, FZD7, CHST7, SLC2A9, ACOX2, KCNK5, ARSE, P2RY1, CSTA, ALDOC, and ANXA8 seem to retain the capability of separating the two lung cancer types. Of note, KCNK5 had been previously proposed in another study [10].

## Discussion

### Candidate genes in lung cancer and other cancer types

We performed a literature search to see if any of the candidate genes (Table 2) had been reported in cancer studies. Mucins will be discussed more in detail below. We discussed below the most interesting results.

The hyaluronan binding protein 2 (HABP2) is an extracellular serine protease, which is up-regulated in LUAD and down-regulated in LUSC has been associated with lung cancer [83]. In lung cancer, the up-regulation of hyaluronan in the extracellular matrix regulate the activity of HABP2 and its regulation of cancer progression has been shown in LUAD, in agreement with our results [83]. Our data and the previous findings suggest that HABP2 may be a valuable target to study for diagnostic and therapeutic purposes.

KCNK5 is a two-pore potassium channel and belongs to the K2P family, i.e., a channel which facilitates the extracellular leak of potassium ions [84]. We found this gene to be up-regulated in LUAD and down-regulated in LUSC. Overexpression of members of the K2P family was associated with different cancer types [84] with some exceptions, such as KCNK4 which was down-regulated. Our results suggest that KCNK5 may be used to classify the two lung cancer types under investigation and that this gene is not necessarily only down-regulated in cancer.

Cystatins, such as CSTA, are cysteine protease inhibitors that regulate different physiological processes [85]. Proteins of this superfamily are classified as tumor suppressors by TSGDB. CSTA is down-regulated in LUAD and up-regulated in LUSC, in our study. CSTA deregulation has been associated with different cancer types [85], and specifically breast cancer. Breast tumors with positive CSTA expression are associated with poor patient outcome [85]. The study on the potential of CSTA as a prognostic marker in LUSC deserves further investigation, along with its regulation by the FOS transcription factor (Fig. 5).

The purinergic receptor P2Y1 (P2RY1) was down-regulated in LUAD but up-regulated in LUSC, according to our study and also identified as a possible LUSC oncogene by *Moonlight*. Of note, its levels can be regulated by the miR-34b-3p microRNA in bladder cancer [86]. Low levels of P2RY1 contribute to the repression of chemoresistance in a concerted action with CCND2 [86] and, in light of our results, it may be an interesting target in LUSC.

Annexin A8 (ANXA8) is down-regulated in LUAD and up-regulated in LUSC, according to our study has been classified as tumor suppressors in TSGDB, suggesting that a similar role in LUAD would benefit of further investigation. At the best of our knowledge, the role of ANXA8 in calcium fluctuation-mediated HIF-1α transcriptional activation and cell viability has been studied only in pancreatic cancer [87].

Frizzled-7 (FZD7) is up-regulated in LUSC, and it is a protein associated with the WNT signaling pathway [88] - a usual suspect in cancer. WNT proteins are secreted glycoproteins, which bind an extracellular cysteine-rich domain of the Frizzled receptor family. FZD7 has been showed as up-regulated in a variety of cancer types including colorectal cancer, hepatocellular carcinoma, and certain breast cancer subtypes [89]. Our data link FZD7 to LUSC. Other genes of the family are classified as possible oncogenes in our study, and the up-regulation of FZD7 would be worth further studies in LUSC since FZD7 is a known pharmacological target. Small peptides or molecules have been reported to inhibit its activity and, as a consequence, suppress the β-catenin-dependent tumor growth [89].

Integrin alpha 6 (ITGA6) is also up-regulated in LUSC in our study and other members of the ITGA family classified either as oncogenes or tumor suppressors (Table 2). Integrins mediate interactions with the extracellular matrix but also drive intracellular communication from the tumor microenvironment leading to migration and invasion. In this context, ITGA6 has been linked to cancer stemness and invasiveness in breast cancer through a HIF-dependent mechanism [90]. Its HIF-dependent up-regulation could be worth exploring in LUSC as well, especially in connection to the link between stemness and resistance to cancer therapy [91].

AQP5 is down-regulated in LUSC and is an aquaporin protein, i.e., a water channel [92]. AQP5 has been reported with a role in invasion in lung cancer, an effect mediated at the protein level and connected with its phosphorylation [92]. The fact that we observed it down-regulated in LUSC, thus, does not imply that its protein level and the protein activity will be affected in this lung cancer type.

FABP5 is a fatty acid-binding protein, which we found down-regulated in LUAD and up-regulated in LUSC. Other FABP proteins have been classified as tumor suppressors or oncogenes, according to our analyses (Table 2), suggesting a complex context-dependent role in cancer. FAB5 has been linked with tumor cell growth, metastasis, and, in certain cases, poor prognosis in other cancer types [93–95]. Therefore, it would be a valuable future direction to explore its role as a marker discriminating LUAD and LUSC in lung cancer tissues.

NRCAM is a neuron-glia-related cell adhesion molecule which is mostly expressed in neurons. Recently, it was also linked to other tissues and cancer types, such as lung adenocarcinoma [96] or thyroid [97]. Our findings are consistent with its down-regulation in LUAD, due to overexpression of CDH2 [96]. On the other hand, NRCAM is clearly up-regulated in LUSC, entailing a promising marker to discriminate the two lung cancer types.

Another interesting candidate is AGR2, which has been associated with lung cancer, as a prognostic marker [98–100]. Our results point to an up-regulation of AGR2 in LUAD, consistently with the findings in literature and down-regulation in LUSC.

#### Genes involved in O-glycosylation of mucins are differentially regulated in different lung cancer types

Our analyses pinpointed a differential regulation of different genes involved in the O-glycosylation of mucins. These genes are up-regulated in LUAD and down-regulated in LUSC.

Mucins are heavily glycosylated proteins where glycosylation is relevant to their function. Under normal conditions, mucins serve as a protective barrier for epithelial lung cells [101]. When dysregulated, these proteins promote cancer progression and metastasis [102]. During cancer progression, mucins can alone or in combination with different tyrosine kinase receptors mediate cell signals for growth and survival of cancer cells. Expression of certain mucins, such as MUC1 or MUC4, have been associated with lung cancer in other studies, and associated with poor prognosis for some patients [102]. Due to this key role in oncogenesis, mucins are emerging as attractive targets for novel therapeutic approaches to treat lung cancer [102].

Our results suggest that both membrane-bound (e.g., MUC21) and secreted mucins (e.g., MUC5B) contributes to the differences between LUAD and LUSC.

Tumors which overexpress MUC5B have been linked to tendencies for relapse and/or metastasize postoperatively in comparison to non-expressing tumors [103]. This finding suggests that LUAD patients could suffer from these events more often than the ones with a LUSC subtype. MUC5B, which we found up-regulated in LUAD and down-regulated in LUSC (Table 2), has also been associated with an aggressive profile in breast cancer. This gene could be targeted to slow down tumor growth and metastasis [104]. Moreover, MUC5B silencing was shown to reduce chemo-resistance of breast tumor cells [105], suggesting this as an interesting target also for LUAD, where we found MUC5B as one of the up-regulated genes. Mucin expression and its link to chemotherapy resistance has been reported even more broadly in cancer [106].

Mucins are amenable drug targets, as attested by MUC1 which can be targeted by immunotherapy thanks to the availability of T-cell specific antigenic epitopes. Vaccines have been proposed, along with aptamer-based drugs (for a review [102]). Despite several studies on mucins in lung cancer, these have only scraped the surface of a complex and intricate interplay where also the interactions between the different mucins can add an extra level of undisclosed complexity. Our results suggest that more studies focusing specifically on MUC5B and MUC21 are needed. The opposite behavior of these two genes in LUAD and LUSC and the overexpression in LUAD suggest the possibility of exploiting them (or the enzymes regulating mucin glycosylation) as drug targets for LUAD-specific therapy.

#### LUSC and the activation of oncogenic pathways for evasion of antitumor activity

Generally, an enhanced immune response in cancer can be exploited for therapeutic purposes [107]. We here observed that LUSC seems to be immune-compromised with a signature of down-regulation of the complement cascade and other key genes for immune response. Our results nicely fit within the overall difference in tumor immune landscape in LUAD and LUSC [108]. To further verify that the differences do not come from differences in the composition of the tumor microenvironment between the two cancer types, we also carried out an exhaustive set of differential expression analyses, correcting for the population of the different cellular infiltrations.

**Fig. 7:**
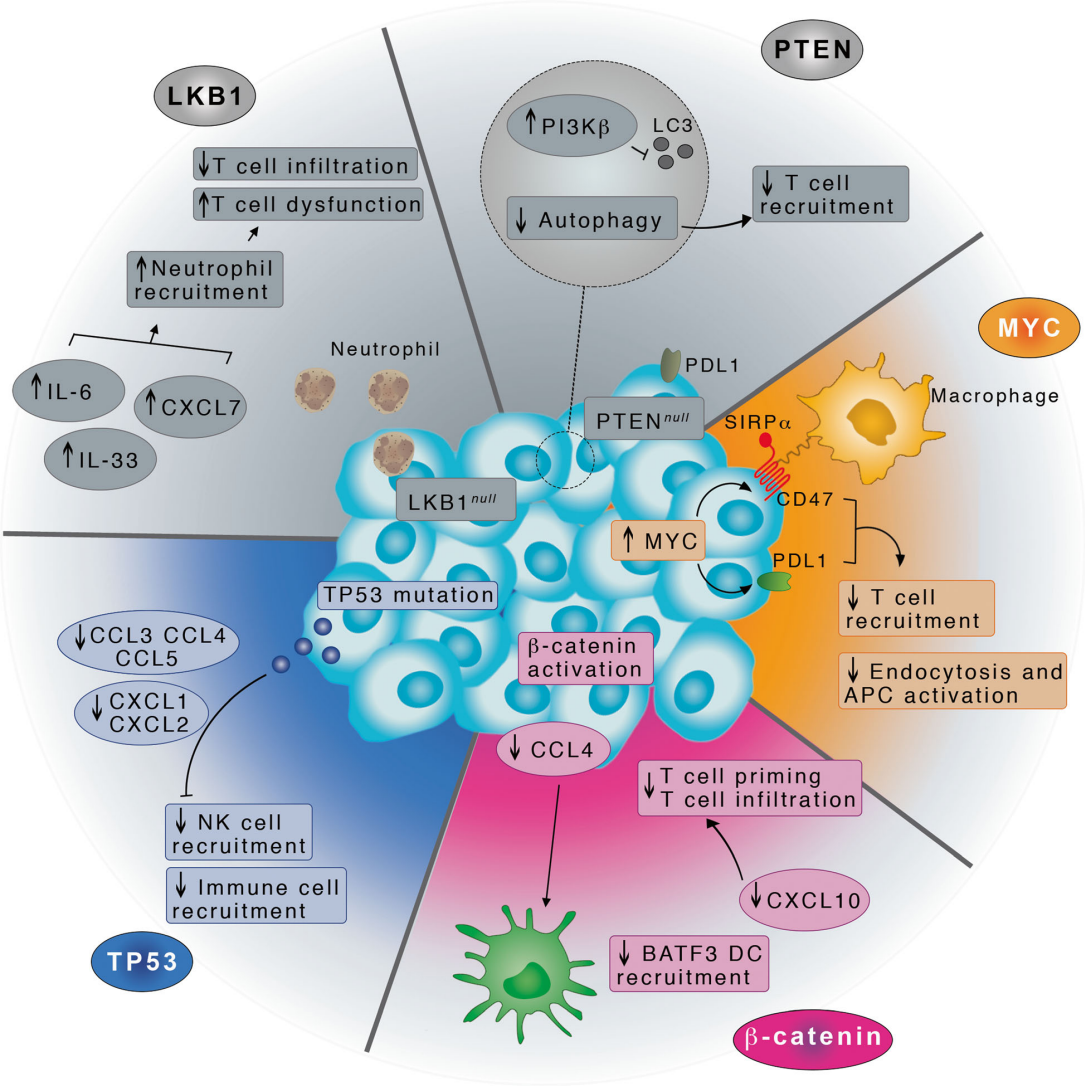
Illustration of the three oncogenic pathways to evade tumor immune response, which could be activated in LUSC. The pathways that are colored in the figure are the ones where the driving down- and up-regulated genes have been found deregulated also in our dataset of DE genes in LUSC. The illustration has been generated with *Adobe Photoshop CC 2014*

Recently, five main oncogenic pathways have been reported [109] that are associated with the evasion of antitumor immunotherapy. The activation of these pathways relies on the dysregulation or mutations of usual suspects in cancer such as p53, cMYC, and the β-catenin/WNT. These genes are the upstream regulators of the evasion pathways and act through a well-orchestrated cascade of other more specific deregulated set of genes (Fig. 7). The oncogenic pathways for evasion of immune response in tumor cells have the ultimate effect of impairing the induction or execution of a local antitumor immune response, which also explains the resistance to certain therapies. We compared the driver up- or down-regulated genes associated to each of these oncogenic pathways [109] with the up- and down-regulated genes in LUSC found in our study. We observed that three evasion pathways (colored in Fig. 7) would be the most suitable candidates to explore further for LUSC, in the direction of the design of new tailored therapies.

## Conclusions

This study allowed to shed new light on the differences between two lung cancer types, i.e., LUAD and LUSC. In addition, we here provided a solid biostatistics and bioinformatics framework for the interpretation of gene expression data. Our data highlight the importance of a careful assessment of protocols for differential expression analyses since too simplistic approaches without the proper information in the design matrix can result in discordant signatures.

We predicted two potential dual role genes (IL6 and KRT23) in LUAD and LUSC. Our analyses also showed that LUAD and LUSC differentiate for the biological processes that are altered. Specifically, LUAD features an up-regulation of genes involved in the O-linked glycosylation of mucins, where MUC5B and MUC21 has the potential for target therapy against LUAD. On the other hand, LUSC seems to be associated with a down-regulation of the complement cascade genes and more generally the innate immune response. These events might be triggered, in LUSC, by the activation of three key oncogenic pathways, stimulated by p53, cMYC and β-catenin that impair the induction of execution of a local antitumor immune response. Future studies on the role of these pathways in LUSC may provide interesting opportunities for drug treatments tailored to this challenging lung cancer type.

We also identified and validated in silico a gene set that could be explored to classify LUAD and LUSC in cancer patient samples. Some of the candidate genes and pathways identified in our study are usual suspects in lung cancer or other cancer types, attesting the validity of our approaches. Moreover, other candidate genes have been poorly investigated, and they could entail novel mechanisms in LUAD and LUSC, deserving attention in future investigations.

## Additional files

Additional file 1: **Text S1** Comparison of different curations of the datasets. The supplementary file includes a detailed comparison of the results achieved using different curations of the TCGA LUAD and LUSC datasets. (DOCX 17 kb)

Additional file 2: **Figure S1** Analysis of the 820 up-regulated genes identified only by *edgeR-TCGAb.* The supplementary figure includes results about the changes in gene expression of the group of up-regulated genes identified only with the old implementation of DEA in *TCGAbiolinks*. (DOCX 1634 kb)

Additional file 3: **Figure S2** GO-enrichment analyses. We reported an example of the results of GO-enrichment analyses for the up-regulated genes in LUAD. (DOCX 391 kb)

Additional file 4: **Figure S3** Additional plot for in silico independent validation of the candidate genes. The heatmap includes the full list of candidate genes using the data from the first validation dataset. See Section 2.10 for more details. (PDF 24 kb)

Additional file 5: **Table S1** Summary of DEGs that have been detected by the different methods or using different dataset curations. The table reports information on the number of up- and down-regulated genes found for each DEA in which we used either a different DEA method or a different curation of the tumor samples. (DOCX 13 kb)

Additional file 6: **Table S2** Integrative annotation for dual role genes. A curation from available datasets of dual role genes. (TXT 2 kb)

Additional file 7: **Table S3** Survival analysis. The table contains all the data from the cox regression on the candidate genes. (XLSX 14 kb)

## Data Availability

The datasets generated and/or analyzed during the current study are available in our Github repository: https://github.com/ELELAB/LUAD_LUSC_TCGA_comparison
