# Supplementary material for: Distinct signatures of lung cancer types: aberrant mucin O-glycosylation and compromised immune response
Source: BMC Cancer. 2019 Aug 20;19:824. doi: 10.1186/s12885-019-5965-x (PMC6702745; doi:10.1186/s12885-019-5965-x)
Supplement: Supplementary file 1 — Text S1 Comparison of different curations of the datasets. The supplementary file includes a detailed comparison of the results achieved using different curations of the TCGA LUAD and LUSC datasets. (DOCX 17 kb) [file 12885_2019_5965_MOESM1_ESM.docx]

**Supplementary Text S1. Comparison of different curations of the datasets.**

We used three different curations of the datasets in the first part of the study: i) two datasets containing all the samples (LUAD_all_, LUSC_all_) that account for partially paired tumors and normal samples; ii) two datasets containing only paired samples (LUAD_paired_, LUSC_paired_), i.e., normal and tumor samples from the same patient; iii) two datasets containing all samples without paired tumor samples (LUAD_unpaired_, LUSC_unpaired_). It has to be noted that some of these datasets are a subset of each other, i.e., samples in the *paired* data sets are fully contained in the *all* the dataset when it comes to both tumor and normal tissues samples. Moreover, the normal samples of the *paired* dataset are fully contained in the *unpaired* dataset and *all* and *unpaired* datasets used the same normal tissue samples.

The pre-processed and processed (after normalization and filtering steps) data used in the study are deposited in our *Github* repository (https://github.com/ELELAB/LUAD_LUSC_TCGA_comparison)**.** A summary of the genes found to be up- or down-regulated by the different combination of curated datasets and DEA approaches is also reported in the *Github* repository, together with the corresponding *UpSet* plots. The *paired* datasets generally aim to account for the removal of individual variability. We noticed that the usage of paired datasets from TCGA made a difference in the comparison of individual gene levels [1] and we thus aimed to evaluate its impact more broadly on our DEA analyses. The usage of the third dataset (*unpaired*) generally aim to remove artifacts due to a partially paired dataset [2] with the purpose of identifying a gene expression profile that may be observed without correction for patient-specific effects. Of note, in our case the overlap between *unpaired* and *all* datasets is large and it is natural that the differences in DEA results are minor. The choice to remove the paired tumor samples was dictated by the fact that the datasets contained several non-paired tumor samples, whereas almost all the normal samples were associated with a paired tumor sample and were thus retained.

The differences in the total number of DE genes were minimal among the different datasets (approximately 1-8 %). We noticed that the usage of paired data provides a stringent selection of up-regulated genes but is more permissive concerning the estimate of down-regulated genes. Moreover, the datasets *unpaired* and *all* feature a more substantial overlap with respect to the *paired* ones, as expected by the fact that they share almost 90% of the samples.

**References**

1. Nygaard M, Terkelsen T, Olsen AV, Sora V, Salamanca J, Rizza F, et al. The mutational landscape of the oncogenic MZF1 SCAN domain in cancer. Front Mol Biosci. 2016;3 December:1–18.

2. Samawi HM, Vogel R. Notes on two sample tests for partially correlated (paired) data. J Appl Stat. 2014;41:109–17. doi:10.1080/02664763.2013.830285.
