# Supplementary material for: Distinct signatures of lung cancer types: aberrant mucin O-glycosylation and compromised immune response
Source: BMC Cancer. 2019 Aug 20;19:824. doi: 10.1186/s12885-019-5965-x (PMC6702745; doi:10.1186/s12885-019-5965-x)
Supplement: Supplementary file 2 — Figure S1 Analysis of the 820 up-regulated genes identified only by edgeR-TCGAb. The supplementary figure includes results about the changes in gene expression of the group of up-regulated genes identified only with the old implementation of DEA in TCGAbiolinks. (DOCX 1634 kb) [file 12885_2019_5965_MOESM2_ESM.docx]

**Figure S1.** **Analysis of the 820 up-regulated genes identified only by *edgeR-TCGAb*.** The comparison between *edgeR-TCGAb* with *limma* and *edgeR*, respectively are shown in the upper panels, whereas the scattered plot comparing *limma* and *edgeR* are shown in bottom left panel. In the upper panels, we reported the genes that: i) are identified as down-regulated (in red); ii) have a significant FDR but not logFC (in green), iii) have a significant logFC but not FDR (in cyan); v) are not significant according to both FDR and logFC (purple), according to *edgeR* (A) or *limma* (B) approaches. In the panel C, we used a similar color code, the only difference was that the condition is satisfied by both the *edgeR* and *limma* approaches (i.e. in red down-regulated genes for both the methods). The genes are in disagreement between *limma* and *edgeR* are shown in grey. We obtained the same results for LUAD and LUSC and we reported the LUAD case as an example.
