# Supplementary material for: Distinct signatures of lung cancer types: aberrant mucin O-glycosylation and compromised immune response
Source: BMC Cancer. 2019 Aug 20;19:824. doi: 10.1186/s12885-019-5965-x (PMC6702745; doi:10.1186/s12885-019-5965-x)

**Figure S2. GO-enrichment analyses.** We illustrate the results from GO-enrichment analyses on the unique LUAD up-regulated genes where it is possible to appreciate the biological processes that are enhanced in LUAD, including the O-glycan processing and cell adhesion processes. The results on O-linked glycosylation are confirmed by the pathway enrichment analyses (see Table 1 main text). We here reported the GO-enrichment analyses on the consensus LUAD DE genes as an example. All the analyses on the other DEA comparisons are reported in the Github repository associated to the publication.


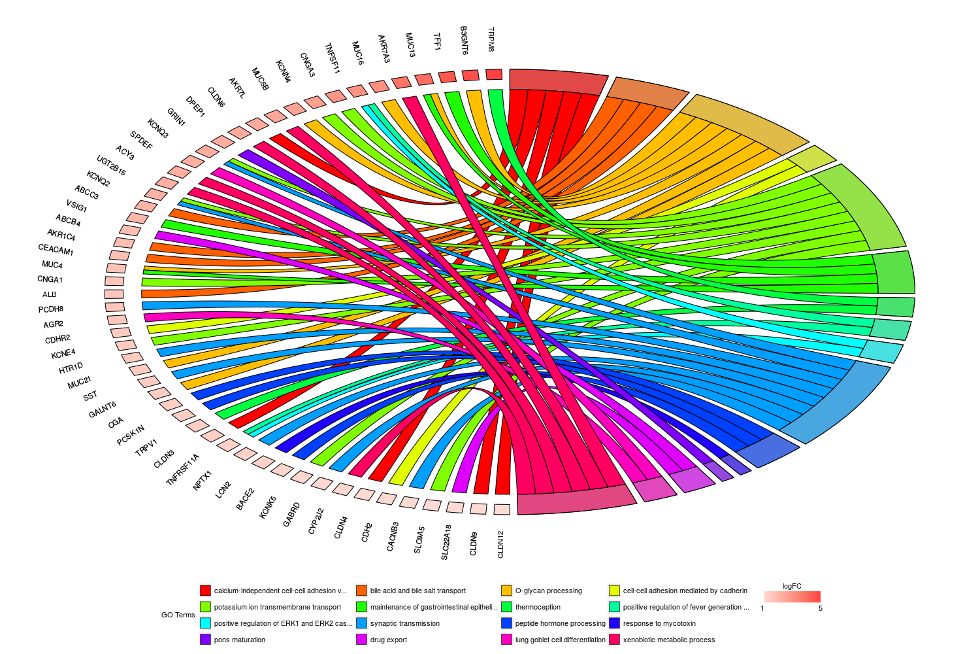

Supplement: Supplementary file 3 — Figure S2 GO-enrichment analyses. We reported an example of the results of GO-enrichment analyses for the up-regulated genes in LUAD. (DOCX 391 kb) [file 12885_2019_5965_MOESM3_ESM.docx]
