# Supplementary figures and images for: Distinct signatures of lung cancer types: aberrant mucin O-glycosylation and compromised immune response
Source: BMC Cancer. 2019 Aug 20;19:824. doi: 10.1186/s12885-019-5965-x (PMC6702745; doi:10.1186/s12885-019-5965-x)

# Color Key

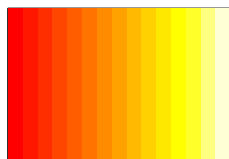

-2      0      2

Value

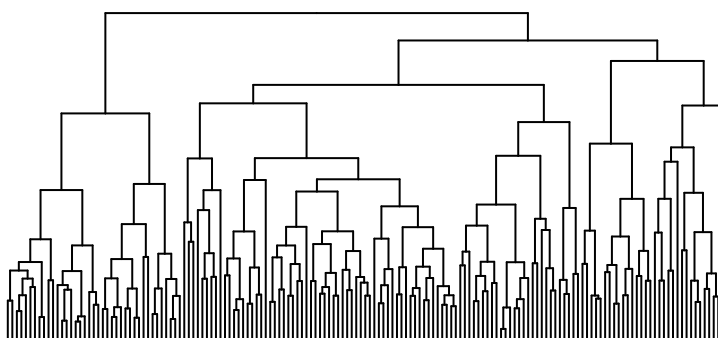

LUAD  
LUSC

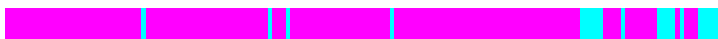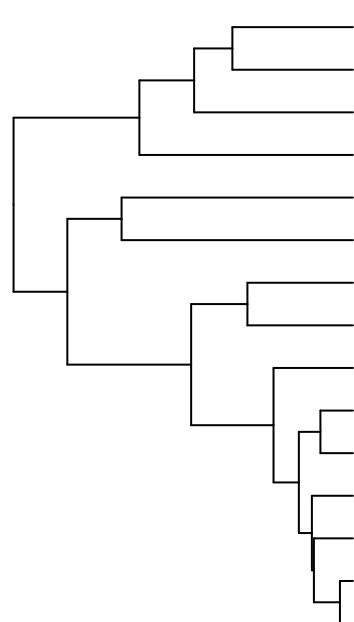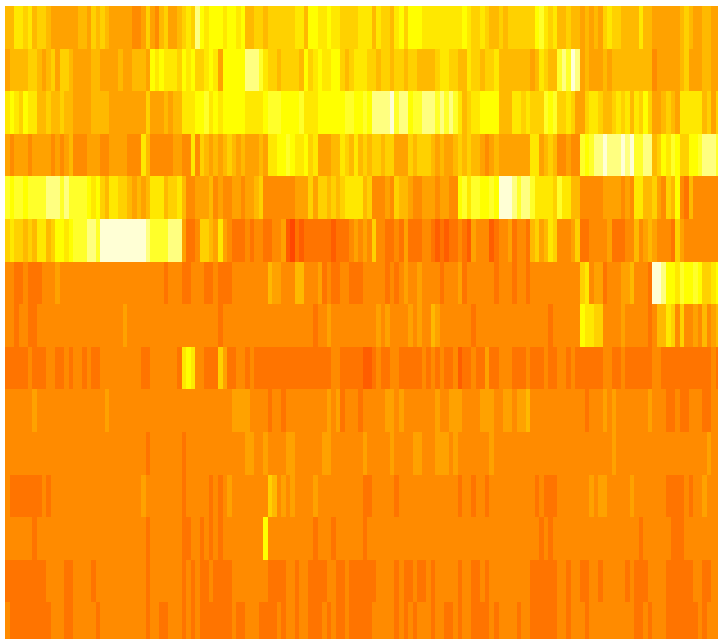

ARSE  
AQP5  
ALDOC  
ITGA6  
AGR2  
MUC5B  
ANXA8  
CSTA  
HABP2  
ICA1  
CHST7  
FZD7  
NRCAM  
ACOX2  
P2RY1

Supplement: Supplementary file 4 — Figure S3 Additional plot for in silico independent validation of the candidate genes. The heatmap includes the full list of candidate genes using the data from the first validation dataset. See Section 2.10 for more details. (PDF 24 kb) [file 12885_2019_5965_MOESM4_ESM.pdf]
