# Supplementary material for: Distinct signatures of lung cancer types: aberrant mucin O-glycosylation and compromised immune response
Source: BMC Cancer. 2019 Aug 20;19:824. doi: 10.1186/s12885-019-5965-x (PMC6702745; doi:10.1186/s12885-019-5965-x)
Supplement: Supplementary file 5 — Table S1 Summary of DEGs that have been detected by the different methods or using different dataset curations. The table reports information on the number of up- and down-regulated genes found for each DEA in which we used either a different DEA method or a different curation of the tumor samples. (DOCX 13 kb) [file 12885_2019_5965_MOESM5_ESM.docx]

**Table S1. Summary of DEGs that have been detected by the different methods or using different dataset curations.**

|  | **edgeR** | | **limma** | | **edgeR_TCGAb** | |
| --- | --- | --- | --- | --- | --- | --- |
|  | **up** | **down** | **up** | **down** | **up** | **down** |
| ${LUAD}_{all}$ | 2176 | 1306 | 1443 | 1859 | 2316 | 1311 |
| ${LUAD}_{unpaired}$ | 2205 | 1358 | 1455 | 1897 | 2326 | 1353 |
| ${LUAD}_{paired}$ | 1263 | 1927 | 1262 | 1882 | 2111 | 1204 |
| ${LUSC}_{all}$ | 3005 | 2111 | 2506 | 2494 | 3161 | 2021 |
| ${LUSC}_{unpaired}$ | 2963 | 2156 | 2485 | 2481 | 3157 | 2039 |
| ${LUSC}_{paired}$ | 2226 | 2667 | 2221 | 2646 | 2854 | 2143 |
